# Health Care Staff’s Experiences of Engagement When Introducing a Digital Decision Support System for Wound Management: Qualitative Study

**DOI:** 10.2196/23188

**Published:** 2020-12-09

**Authors:** Hanna Wickström, Hanna Tuvesson, Rut Öien, Patrik Midlöv, Cecilia Fagerström

**Affiliations:** 1 Department of Clinical Sciences Malmö Center for Primary Health Care Research Lund University Malmö Sweden; 2 Blekinge Wound Healing Centre Karlshamn Sweden; 3 Department of Health and Caring Sciences Linnaeus University Växjö Sweden; 4 Blekinge Centre of Competence Karlskrona Sweden; 5 Department of Health and Caring Sciences Linnaeus University Kalmar Sweden

**Keywords:** decision support systems, clinical, eHealth, staff engagement, leg ulcer, telemedicine, mobile phone

## Abstract

**Background:**

eHealth solutions such as digital decision support systems (DDSSs) have the potential to assist collaboration between health care staff to improve matters for specific patient groups. Patients with hard-to-heal ulcers have long healing times because of a lack of guidelines for structured diagnosis, treatment, and follow-up. Multidisciplinary collaboration in wound management teams is essential. A DDSS could offer a way of aiding improvement within wound management. The introduction of eHealth solutions into health care is complicated, and the engagement of the staff seems crucial. Factors influencing and affecting engagement need to be understood and considered for the introduction of a DDSS to succeed.

**Objective:**

This study aims to describe health care staff’s experiences of engagement and barriers to and influencers of engagement when introducing a DDSS for wound management.

**Methods:**

This study uses a qualitative approach. Interviews were conducted with 11 health care staff within primary (n=4), community (n=6), and specialist (n=1) care during the start-up of the introduction of a DDSS for wound management. The interviews focused on the staff’s experiences of engagement. Content analysis by Burnard was used in the data analysis process.

**Results:**

A total of 4 categories emerged describing the participants’ experiences of engagement: *a personal liaison*, *a professional commitment*, *an extended togetherness*, and *an awareness and understanding of the circumstances*.

**Conclusions:**

This study identifies barriers to and influencers of engagement, reinforcing that staff experience engagement through feeling a personal liaison and a professional commitment to make things better for their patients. In addition, engagement is nourished by sharing with coworkers and by active support and understanding from leadership.

## Introduction

### Background

Most patients with hard-to-heal ulcers, defined as ulcers that take more than 4 weeks to heal [1], are older adults and have multiple diseases [2]. Unfortunately, many patients with such ulcers are treated without diagnosis and receive suboptimal treatment, thus prolonging healing time [3]. The lack of national guidelines, decision support systems, and structural organization for this patient group is common in Sweden, making wound management difficult for patients and staff. Wound management is carried out by different caregivers within different medical specialties, and a multidisciplinary team of professionals is often necessary to establish the ulcer etiology and to provide diagnosis [4] and treatment. In Sweden, most patients with hard-to-heal ulcers have their continuous treatment in primary and community care [3], where nurses and physicians need to cooperate in wound management teams. The assigned nurse meets the patient approximately twice a week for ulcer treatment and dressing changes. The physician meets the patient for diagnosis and for the decision of referral to other clinical specialties such as vascular intervention. The assigned nurse is often responsible for the continuity of care and initiates contact with the physician when needed. Patients with hard-to-heal ulcers are generally diagnosed through in-person assessment, but a few studies have discussed the advantages of using eHealth solutions in wound management [5]. For this patient group, eHealth is expected to enable medical investigation and treatment for healing already in the home environment, to reduce transportations to different caregivers, and to minimize hospitalization [5,6].

eHealth solutions have been introduced in health care in recent years to increase accessibility and facilitate diagnosis and care [5,7]. eHealth is defined as the use of information and communication technologies for health [8] and includes various forms of digital transmission of imaging and clinical data. Digital decision support systems (DDSSs) are a type of eHealth solution designed for clinical decisions and medical education [9] and for facilitating a multidisciplinary working environment and quality-assured guidance [10,11]. Few studies have focused on health care staff’s engagement and barriers to and influencers of engagement during the introduction of eHealth interventions such as DDSSs, although the aspects of engagement are often described as crucial for the introduction and implementation [12,13] of new ways of working.

Some of the existing literature defines engagement as a psychological process relating to user experiences and perceptions, whereas other literature defines engagement only as intervention usage [12-14]. This discrepancy can be explained by the different disciplines involved in the interventions: medical, social, psychological, or technological [15]. An expanded definition of engagement with eHealth interventions might include the extent of usage (eg, amount, frequency, duration, and depth) and the subjective experience characterized by attention, interest, and enjoyment [13]. More narrow descriptions of engagement involve active support for a project [16] or when an employee is enthusiastic about their work and takes a positive initiative to promote the organization’s interests. To sum up, the process of engagement is a multidimensional experience characterized by cognitive, emotional, and behavioral dimensions [17]. In this context, engagement is conceptualized by health care staff’s expressions of experience, not frequency of usage. Attempts to understand engagement in new ways of working within health care are also made more difficult, as engagement is often described only from the patient perspective [17,18]. Very few studies describe health care staff’s engagement in eHealth interventions, and all these consider the perspective of one specific profession [19,20]. It is therefore of interest to take a broader perspective and investigate the engagement of several different types of health care staff: physicians, nurses, and assistant nurses, all of whom are part of a wound management team in primary, community, or specialist care. Methods to measure and evaluate engagement vary greatly but include interviews, self-report questionnaires, verbal reports, automatic recordings of use, and recordings of psychophysical manifestations [12,15].

There are indications that the introduction and implementation of new eHealth solutions into everyday work practice is complicated [21], and only a few such solutions have become useful in clinical practice. One explanation for this may be that eHealth solutions are not always harmonized with the specific context in which they are to be implemented, including the prevailing organizational cultures, values, and routines [6]. Nurses associate the introduction of eHealth in homecare with the risk of deprofessionalization [22], and studies have also described a fear that if the new technology lacks sufficient usability, mistakes might occur that endanger patient safety [23]. The importance of leadership [21,24] and a shared vision with coworkers [24,25] have been pointed out as influencing factors for successful implementation. Studies highlight that unless users are involved in the design process of an eHealth solution [7,26], the implementation process will be ineffective. Lack of time within daily clinical routines is also described as a barrier in the implementation process [21]. All the aforementioned barriers and influencers might also affect staff engagement and thereby complicate implementation further. To implement new working processes such as eHealth solutions, it is essential to gain knowledge concerning staff’s experiences of engagement and to create conditions for long-term engagement.

### Objectives

The aim of this study is to describe health care staff experiences of engagement and barriers to and influencers of engagement when introducing a DDSS for wound management.

## Methods

### Study Design

A qualitative interview design was selected to describe the health care staff’s experiences of engagement. The study followed the guidelines presented in the COREQ (Consolidated Criteria for Reporting Qualitative Research) framework [27].

### Setting

Launched in 2009, the Swedish National Quality Registry of Ulcer Treatment (RUT) is a tool for clinical assessment of hard-to-heal ulcers [28]. In collaboration with a technology company, it has developed a DDSS, known as Dermicus Wound (Multimedia Appendix 1), to help wound management teams in primary, community, and specialist care establish collaboration for ulcer diagnosis and treatment. The DDSS offers easily downloadable mobile apps for bedside automatic transmission of mandatory data to the RUT and to a platform for multidisciplinary consultation with the ability to share medical information and photographs in wound management teams. The DDSS is designed to be used when a nurse or assistant nurse meets a patient with a hard-to-heal ulcer for the first time. The standardized data for ulcer diagnosis, such as age, gender, smoking habits, ulcer duration, ulcer size, ulcer pain, ankle-brachial index, comorbidities, and photographs of the ulcer and dressing materials, are collected by the nurse or assistant nurse and transmitted from the app to a platform. An email is sent, as an alert, to a chosen connected participant within the nurse’s wound management team that a new case has been received. The connected participant can then enter the platform to assess the data and photographs and recommend treatment strategies to the submitting staff. The wound management team can also invite external consultants to advise on especially complex patient cases. The DDSS is designed to delete all data and photographs from the app following transmission to the platform. The DDSS is Conformité Européenne certified according to medical devices class I (D3.0-112015) and compliant with health care regulations and the General Data Protection Regulation. It is compatible with the iPhone with the standard touch screen user interface and camera installed and with standard web browsers.

The DDSS was launched on RUT’s website during an annual user meeting for registrars. In total, 65 health care staff from primary, community, and specialist care agreed to test the DDSS for 6 months. All participants were invited to participate in this interview study, and 11 agreed.

### Participants

All the participants (n=11) frequently treated patients with hard-to-heal ulcers. Their workplaces had a wide geographical spread from southern to northern Sweden, including both urban and rural areas. Some of the participants had a managerial position or worked as coordinators for wound management. The participants working in primary care (n=4) were 2 general practitioners (1 female and 1 male) and 2 female nurses. The participants working in community care (n=6) were 3 female nurses, 2 male nurses, and 1 female assistant nurse. The final participant was a female assistant nurse who worked in a specialist clinic (n=1). Thus, 8 participants were females and 3 were males. The technology company demonstrated the DDSS to the participants before testing, but no further organized training was provided. All participants were given the same information and technical support, and the DDSS was free to use during the test period of 2018 to 2019. Before the interviews were conducted, the participants were informed orally and in writing about the study, confidentiality, and voluntary nature of participation. All participants provided signed consent. The Ethical Advisory Board in South East Sweden reviewed the project (ref: 506-2018).

### Data Collection

The participants used the DDSS over a testing period of 6 months and underwent an individual semistructured telephone interview based on questions about their engagement in the introduction of the DDSS. The interviews were conducted within the first month of the introduction and were carried out between October 2018 and May 2019. An interview guide was used. The questions were open and started with a general question about the concept of engagement: “What do you think of when you hear the word ‘engagement’ in relation to changes in work processes?” The following questions were about the participants’ individual engagement in relation to the DDSS, for example: “How do you experience your engagement in the DDSS?” with supporting questions, such as, “Can you elaborate?” and “Can you tell me more about this?” The interviews lasted between 29 and 48 min and were recorded and transcribed verbatim. The interviews were led by 1 of the 3 moderators: HW, HT, and CF. HW works as a physician at a wound healing center for patients with hard-to-heal ulcers, whereas HT and CF are conducting ongoing research within the engagement process, which constitutes their preunderstanding.

### Data Analysis

The data were analyzed according to the Burnard method for qualitative content analysis [29,30]. The content analysis was performed in an inductive manner [31]. The recorded interviews were listened to at least four times each. The moderators read the transcribed interviews and field notes individually. With the aim of the study in mind, notes were written down in the margin of the interview text and field notes. These notes were distributed among the moderators. The text was divided into units of meaning comprising sentences and paragraphs and then condensed while preserving their core. Codes were identified in the condensed text; these were compared with the original transcribed texts and field notes to ascertain whether the context was maintained by the codes. The codes were put into a matrix and then compared and ordered into subcategories. Similar subcategories were combined into categories. To increase validity, the 3 moderators analyzed the text separately and then compared and discussed their listed units of meaning, codes, subcategories, and categories. The moderators re-read the transcribed interviews to ensure that the categories and subcategories reflected what had been said in the interviews [29].

## Results

### Overview

A total of 4 categories emerged from the analysis, reflecting these health care staff’s experiences of engagement when introducing a DDSS for wound management: *a personal liaison*, *a professional commitment*, *an extended togetherness*, and *an awareness and understanding of the circumstances*. Each category had 2 subcategories (Textbox 1).

**Categories and subcategories based on the participants’ experiences of engagement.**

- A personal liaison:
  Being vigorous and passionate
  Seeking new knowledge and self-development
- A professional commitment:
  Making things better for a neglected patient group and for society
  Striving for safe and structured care
- An extended togetherness:
  Anchoring with coworkers and patients
  Connecting through support and inspiration
- An awareness and understanding of the circumstances:
  Being directed by a supportive leadership
  Considering time and timing

### A Personal Liaison

The first category, *a personal liaison*, reflected how the participants expressed engagement as a constituent of their personality and their seeking for self-development via new knowledge. The basis for engagement came from themselves and their own personal attitudes, hopes, and driving forces. The personal liaison seemed to be essential for a successful implementation and relied on positive attitudes toward future improvements together with continuous seeking for improved skills. Feeling and having a personal liaison with the intervention was described as a positive influencer of staff engagement.

#### Being Vigorous and Passionate

When asked what engagement meant to them, the participants highlighted their underlying personalities and described themselves as vigorous, passionate, driven, and enthusiastic about future changes. This was affirmed in terms of a personal liaison. The participants described their strong capacity to initiate work changes and stated that a vigorous personality was a positive influencing factor for engagement:

If I think something is good, I don’t think I’ll let anything stop me.

Participant 6

The participants expressed engagement in positive terms, saying that they were excited about and looked forward to the challenge of staying up to date with technology and modern treatment methods. They experienced increased engagement when personally taking part in the development of a new eHealth solution and were excited to evaluate how it could be used within health care. The participants’ engagement was also influenced by whether or not they were personally affected in the introduction of a new working method, for example, if the new work tool facilitated their work and gave them a direct personal gain or if they just used the DDSS in passing.

#### Seeking New Knowledge and Self-development

In addition to describing themselves as having a vigorous and passionate personality, the participants stated that they were engaged by the quest to acquire new knowledge, giving them a chance for enhanced competence and confidence within a specific area. Engaging in seeking new knowledge through life was a way of self-development for their own sake and for their own self-esteem. An interest in seeking new knowledge produced and nourished the personal liaison of engagement:

I saw it as a huge opportunity, both for myself and for the workplace, to ... to, like, develop in this, both for my own part, like, for myself, just because I think it’s ... I think it’s fun to gain new knowledge and to get better at things and so on.

Participant 11

The pursuit and wish for new knowledge were expressed as fundamental for engagement in work changes. The participants described a need for increased competence and education in wound management and believed that the DDSS would help with this. The participants described how responses from a coworker or consultant on the shared digital platform increased their own skills and knowledge of how to handle similar cases themselves. Evidence-based knowledge was mentioned as desirable and as a strong, engaging factor. The fact that a national quality registry was backing the DDSS gave confidence and an assurance of evidence-based and quality-based knowledge.

### A Professional Commitment

The second category covered experiences of *a professional commitment* emerging as a positive influencing factor for the participants’ engagement. The participants experienced that the reason and power to become engaged in the DDSS came from their professional commitment to do good for their patients, as the patients were the core value for doing anything. This, in turn, emanated from an obvious need for safe and structured medical care for this specific underprioritized patient group.

#### Making Things Better for a Neglected Patient Group and for Society

The participants described patients with hard-to-heal ulcers as being neglected and not prioritized in health care. They spoke about a lack of continuity and quality, and this obvious medical need positively influenced engagement in the introduction of the DDSS:

If you see an area where an improvement is needed, where there’s developmental work that needs to be done. That’s something that fosters engagement.

Participant 1

The participants felt that a prerequisite for engagement was the belief that the DDSS was directly beneficial to their patients. For them, the most important issue was to make things better for the patients, and this was why they would engage. The participants supposed that the DDSS could lead to reduced suffering, faster healing times, and fewer transportations for patients with hard-to-heal ulcers. The participants were engaged by the expectation of being able to provide better service and equal health care to the patients regardless of where they lived, that is, the patients could be treated at home, especially patients living in the countryside far from health care. Another factor positively affecting their engagement was the ability to use eHealth to avoid exposing patients to unnecessary examinations and surgeries, referring to the possibility of dismissing suspicions of malignancy in the ulcer by having a photograph assessed. The participants received encouraging responses from pleased patients and patients’ relatives, which was interpreted as an acknowledgment of better care; this again positively affected engagement. Conversely, if patients were unwilling to use the DDSS, this would directly impose a barrier to continued engagement and then they would have to find other ways to aid improvement.

Additionally included in this subcategory were factors such as participants’ engagement in doing good for society and in saving taxpayers’ money by becoming more efficient in wound management. Nevertheless, in the first place, engagement was described as increasing due to the professional commitment to make things better for the individual patient and to make this patient group and its medical need more visible:

I’m not providing care to some financial system or a budget or something like that—it’s supposed to benefit an individual person who is ill.

Participant 8

#### Striving for Safe and Structured Care

The participants described the current wound management as unstructured and unsafe. One part of the participants’ professional commitment was to give patients with hard-to-heal ulcers a clear structure in their medical care; this target initiated their engagement. The participants were engaged by a belief that the DDSS could provide structure in the organization and secure new ways of communication to improve efficiency. They expressed that the DDSS could gather and organize a few involved nurses and physicians in local wound management teams for diagnosis and treatment with continuity, thereby increasing the quality of care:

Fundamentally, I guess it’s about getting a structure and, like, building up a good wound healing clinic here at our primary health care centre.

Participant 4

The participants also mentioned technical safety and usability as influencing factors of engagement. They pointed out that the DDSS had an uncomplicated and well-known technology that created a safer structure in clinical praxis. The fact that clinically active staff were involved in designing the DDSS and adapting it to clinical reality created engagement and security. The participants experienced that their engagement was positively influenced by the possibility of using a technically secure way of communicating with other caregivers:

If we can find a system in which I can securely transfer information to a colleague in another organization, that would be fantastic.

Participant 8

Technical difficulties were mentioned, such as the lack of a spellchecker, sufficient space on the smartphones, immediate access to the patients’ medical records, and compatibility with Android technology. Other technical problems included disruption and double documentation. These technical obstacles were described as risk factors for patient safety and hence as barriers to engagement, as protecting patients from risks was a part of the professional commitment.

### An Extended Togetherness

The third category, *an extended togetherness*, described how the interaction between and within the wound management teams, with patients, other coworkers, external consultants, and the project team was essential for participants’ engagement when introducing the DDSS. A feeling of togetherness around the patients increased and spread engagement, like a fabric for collaboration, and was a prerequisite for using the DDSS.

#### Anchoring With Coworkers and Patients

In order for the participants to maintain engagement with and enthusiasm for the DDSS, it was necessary for many coworkers and even patients to be engaged. Another prerequisite for engagement was a broad anchoring and encouragement within the participants’ own group of coworkers. Support from coworkers was described in terms of positive traction, being grateful not to be alone, and having a feeling of togetherness. The participants expressed a wish that more coworkers felt the same engagement and said it was important to convince everyone that this new work process offered a chance for all of them to achieve progress in wound management. For example, the participants described how if a nurse did not have medical support from a physician, the nurse found it difficult to dare to try the DDSS. However, when there was medical support from a physician, the nurse felt safe in collaborating, and everything went well. In addition, participants who responded on the platform expressed great engagement in delivering answers promptly:

I feel a lot of engagement—it’s like I want to respond if they write questions, because I see a great benefit there.

Participant 3

The participants expressed that they could support each other within the team; if one person’s engagement decreased, someone else could bring up engagement again. If coworkers opposed and questioned continuously, that would be a barrier to engagement:

If you don’t have the working team with you, if you feel like you’re being obstructed, those can be the kinds of things that counteract your engagement.

Participant 9

Another engaging factor was the opportunity to make patients more involved and engaged in their own treatment because of the possibility of visually following the healing process in the DDSS. The participants highlighted that when introducing new tools, health care staff and patients must interact, creating togetherness.

#### Connecting Through Support and Inspiration

The participants experienced that support from external consultants and inspiration from the project team positively influenced engagement. They felt engaged by the opportunity to be connected to a specialist in wound management, to get feedback on their work, and to get support when assessing diagnoses and providing the best treatment. Stress and frustration were described to be reduced by knowing that there was someone to consult, who could give support and direct the participants toward the right clinical decision. Cooperation across the boundaries of different caregivers was described as an existing obstacle in Swedish health care, despite being a necessity for this patient group. The participants expressed hope that the DDSS would bridge this gap, create new connections, and extend engagement to other clinics. This expectation of extended interaction and togetherness around the patients fostered further engagement. A user meeting for health care staff registering in the RUT, conducted before the start of the DDSS introduction, was expressed to positively influence engagement and give inspiration, connecting participants into a network where engagement was shared. Meeting the project team face-to-face on this occasion facilitated efficient digital support during the introduction:

They gave a lot and in some way that increased the engagement that was already there, or, like, you felt that no, but ... that you ... that you were a bit more inspired to get going.

Participant 4

### An Awareness and Understanding of the Circumstances

The final category covered considering factors for engagement, such as the leadership’s *awareness and understanding* of what environmental and contextual resources were needed for a successful introduction of the DDSS. Lack of working time, lack of resources, and the timing of the introduction were potential barriers to participants’ engagement.

#### Being Directed by a Supportive Leadership

Leadership awareness and understanding of the introduction colored and influenced the participants’ engagement. The participants considered it important for the leadership in their own organization to be supportive, enthusiastic, and willing to arrange technical resources:

The manager, she’s very positive about this as well, and of course that makes it easier when you’re doing something that involves the entire team and so on.

Participant 10

One manager brought 3 smartphones to the wound management team at the beginning of the DDSS introduction; this made the participants feel that introducing this working tool was highly prioritized and important, which positively influenced engagement. Conversely, when the leadership showed no understanding of what resources were required to make the DDSS available, the participants’ engagement was negatively influenced. The participants expressed that leadership should show confidence in the ability of engaged employees to plan and run the introduction of new eHealth solutions. The participants experienced a negative impact on engagement if the idea of introducing eHealth solutions was top to bottom:

It can come from the top, from the administration ... where we don’t really, like, see the needs, and then it won’t be something ... that also counteracts the engagement.

Participant 2

#### Considering Time and Timing

The lack of working time and timing were disadvantageous circumstances for participants’ engagement when introducing the DDSS:

One thing that counteracts engagement, is the lack of time, that ... this compassion and participation ... can fail because you don’t have the time to do things in the way that you would always like to.

Participant 5

Time was described as crucial for engagement. The lack of working time was experienced as a barrier to engagement and thus described as a source of stress and frustration. In addition, the DDSS itself could be time consuming, which negatively affected engagement. A stressful working environment meant that the participants could not always prioritize using the DDSS, although they recognized that a system like this would have been most valuable for the patients. The participants expressed hope that in the long run, the DDSS would generate faster and more effective consultation, thereby saving time. The timing of the introduction also influenced engagement. If the introduction took place during stressful circumstances, engagement decreased, but if the DDSS was introduced in a more structured and calmer period, engagement increased. During summertime or relocation, it was difficult and impracticable to introduce new working processes because of fewer employees and a heavier workload.

## Discussion

### Principal Findings

The participants in this study described barriers to and influencers of engagement in the introduction of a DDSS, resulting in 4 categories: *a personal liaison*, *a professional commitment*, *an extended togetherness*, and *an awareness and understanding of the circumstances*. The principal findings are that engagement arises when health care staff do something meaningful for themselves and their self-development, in combination with the professional commitment to improve things for this patient group. In addition, the staff need to feel togetherness with their surroundings (ie, the wound healing team, coworkers, and patients) and to have support and understanding from leadership regarding the resources needed, such as extra working time, timing, and equipment. These aspects of the staff’s experiences of engagement in the introduction of a DDSS for wound management can provide guidance when building and introducing future eHealth solutions in health care.

The participants experienced engagement as a personal liaison, describing themselves as having a vigorous and passionate personality that formed the basis for engagement in new working processes. The influence of personal factors such as optimism and self-efficacy on engagement has been previously shown in a literature review of nurses’ work engagement [20]. Furthermore, the participants experienced that the personal liaison was built upon their own interest in becoming better, striving for new knowledge and skills within a specific medical field. This is in line with a systematic review conceptualizing one part of engagement to be a subjective experience based on the individuals’ attention, interest, and affect [13]. The same systematic review [13] described personal relevance and expectations as influencers of engagement, which could also be seen in this study. The participants perceived that the DDSS applied to their individual situation when treating patients with hard-to-heal ulcers, and this personal relevance positively influenced engagement. The expectation that the DDSS could make things better for their patients was emphasized as a prerequisite for engagement. Thus, what engaged the participants goes hand in hand with the aim of introducing DDSS into health care, that is, to promote medical education and improve clinical skills [6,9]. Hence, a DDSS needs to catch health care staff’s personal interest and attention and contribute to improved skills and knowledge to make them engaged.

Patients with hard-to-heal ulcers constitute an older adult and fragile patient group. The present results clearly show that the benefits of introducing eHealth to this patient group were obvious and engaging for the participants. According to an earlier comprehensive Swedish government review, the staff strive for increased structure in wound management organizations [2], which seems to be in line with what made the health care staff in this study engaged in the DDSS. The participants expressed engagement by providing structured and patient-safe treatment in the patients’ home environment, which was possible to do when using the DDSS. Studies have shown that older adults and chronically diseased patients themselves can see the potential of eHealth to help them continue living in their own homes [32,33], and this is in accordance with what made the health care staff in this study engaged in being able to offer. The participants experienced engagement and confidence in the DDSS if their patients were pleased; in contrast, they said that if patients expressed dissatisfaction with the DDSS, then this would negatively influence their engagement. One study found that physician engagement decreased because they believed that the use of a bedside DDSS was seen as unprofessional by patients [34]. The patients, on the other hand, perceived the use of a bedside DDSS as positive, signaling confidence and competence [34]. Patients’ positive views of eHealth solutions derive from their feeling that these solutions allow the staff to gain knowledge and expertise [18,20], and, clearly, patients’ opinions color staff engagement.

An earlier study described the importance of DDSS being adapted to both local and national guidelines, showing that this influenced engagement when physicians tried a DDSS in clinical praxis [34]. The participants in our study did not express any fears that the DDSS might be unsuitable or incompatible with any guidelines or the context; instead, they felt safe using it for the actual patient group. This is in contrast to previous findings where nurses felt afraid of losing context [6] or had feelings of deprofessionalization [22] when introducing eHealth tools.

Technical problems were reported as barriers to engagement. This is in accordance with findings from other studies on introducing eHealth solutions in health care [13,34]. The DDSS in this study used a well-known technical system that was easy to use, which seemed to balance this out to some extent, but there was still a risk of disengagement. The fact that clinically active staff and the quality registry were involved in designing the DDSS provided participants with assurances of safety, increasing engagement. This is in agreement with earlier findings that users’ confidence increased if they had been involved in the development of the eHealth solution [7,26] and a study where staff described it as important for decision support systems to offer a sense of professional security [35]. Technical support by the project team was provided in an efficient way, which positively influenced participants’ engagement. This is consistent with another study where the lack of technical support was described as a major barrier to engagement with technology solutions in health care [34].

The togetherness within the team, with other coworkers, and with patients seemed to be crucial for participants’ engagement. It was essential to feel part of a supportive and encouraging environment where engagement could spread, bringing teams together, which, in turn, nourished further engagement. The participants strived for and desired to extend engagement to other clinics for broader collaboration. This togetherness decreased the feeling of loneliness and positively influenced participant engagement, as shown earlier in a systematic review of engagement in digital behavior change interventions [13]. Another literature review [20] also found that feeling part of a community and having social support increased nurses’ engagement in work. Between the lines, there was a desire to feel affirmation, acceptance, and togetherness from others, that what they do is good. Hard-to-heal ulcers require the participation of physicians, nurses, and assistant nurses, and the inclusion of all these actors in the DDSS enables necessary collaboration and creates togetherness, which positively influences engagement. The importance of multidisciplinary collaboration has been described in a systematic literature review of treating chronic wounds by using decision support systems [36]. This review pointed out that most existing systems were built to meet the needs of nurses or physicians separately, which would be contrary to multidisciplinary collaboration [36]. There is a need for future studies focusing on the interaction between staff and its importance for engagement.

The staff in this study described how distinct and supportive leadership positively influenced engagement. Many previous studies have highlighted and confirmed the importance of leadership for successful eHealth implementation [20,21,24,37]. One key to the successful introduction of new working processes might be the leadership’s ability to create conditions where staff engagement can thrive and persist. The participants expressed it as important for leadership to provide resources, especially working time, but also to plan the timing of the introduction and ensure that the necessary equipment was purchased. The fact that resources are required for engagement has been shown in studies of engagement in organizational improvements [38] and in digital behavior change interventions [13]. The most frequently described resource is working time, and many earlier studies have shown this to be important for engagement and implementation [6,21,34,37]. However, most important for the participants in this study was that the leadership showed an understanding of the new working process as well as confidence in allowing the engaged staff to lead the introduction. Many studies have highlighted time [6,21,34,37] and leadership [20,21,24,37], indicating that these 2 parameters are crucial for both engagement and introduction; they constitute basic premises that must be functioning and assured before the introduction. However, the apparent importance of this may also be because there are limited studies of other factors influencing engagement. The focus of studies on the successful introduction of eHealth in health care might need to be broadened, including personal and professional factors affecting staff engagement and their significance, in addition to resources, support, and understanding from the surroundings.

### Methodological Considerations and Limitations

The data were presented objectively, as none of the moderators were involved in using or launching the DDSS, thus assuring confirmability. Concerning credibility, the moderators listened to and read all the interviews to ensure that no relevant data had been excluded. To ensure dependability, memos were used to track changes in the coding decisions and hence keep track of recoding and relabeling. The participants were representative of wound management, with all different disciplines involved, and with a vast geographical spread throughout Sweden, making the results transferable*.* The participants were both female and male. All participants received similar information about the interviews. The interviews were conducted in real time during the introduction, instead of afterward, which is another strength of the study. The fact that the participants volunteered to participate in the study is a limitation, as the staff who did not volunteer may have been those who were not engaged in the DDSS. Finally, the experiences of engagement belong to the participants themselves and hence can only to a certain extent be compared with other research.

### Conclusions

This study contributes to the awareness of aspects that need to be considered in relation to engagement when introducing a DDSS for wound management. The findings indicated that staff experience engagement through feeling a personal liaison and a professional commitment to make things better for patients with hard-to-heal ulcers. In addition, their engagement needed nourishment by sharing with coworkers and by active support and understanding from leadership. Future research needs to explore potential obstacles to long-term engagement, as this study only included the time of introduction.

## Appendix

Multimedia Appendix 1 A photograph of the digital decision support system, the Dermicus Wound.

## Abbreviations

COREQ: Consolidated Criteria for Reporting Qualitative Research
DDSS: digital decision support system
RUT: Registry of Ulcer Treatment
