# Supplementary figures and images for: Health Care Staff’s Experiences of Engagement When Introducing a Digital Decision Support System for Wound Management: Qualitative Study
Source: JMIR Hum Factors. 2020 Dec 9;7(4):e23188. doi: 10.2196/23188 (PMC7758170; doi:10.2196/23188)

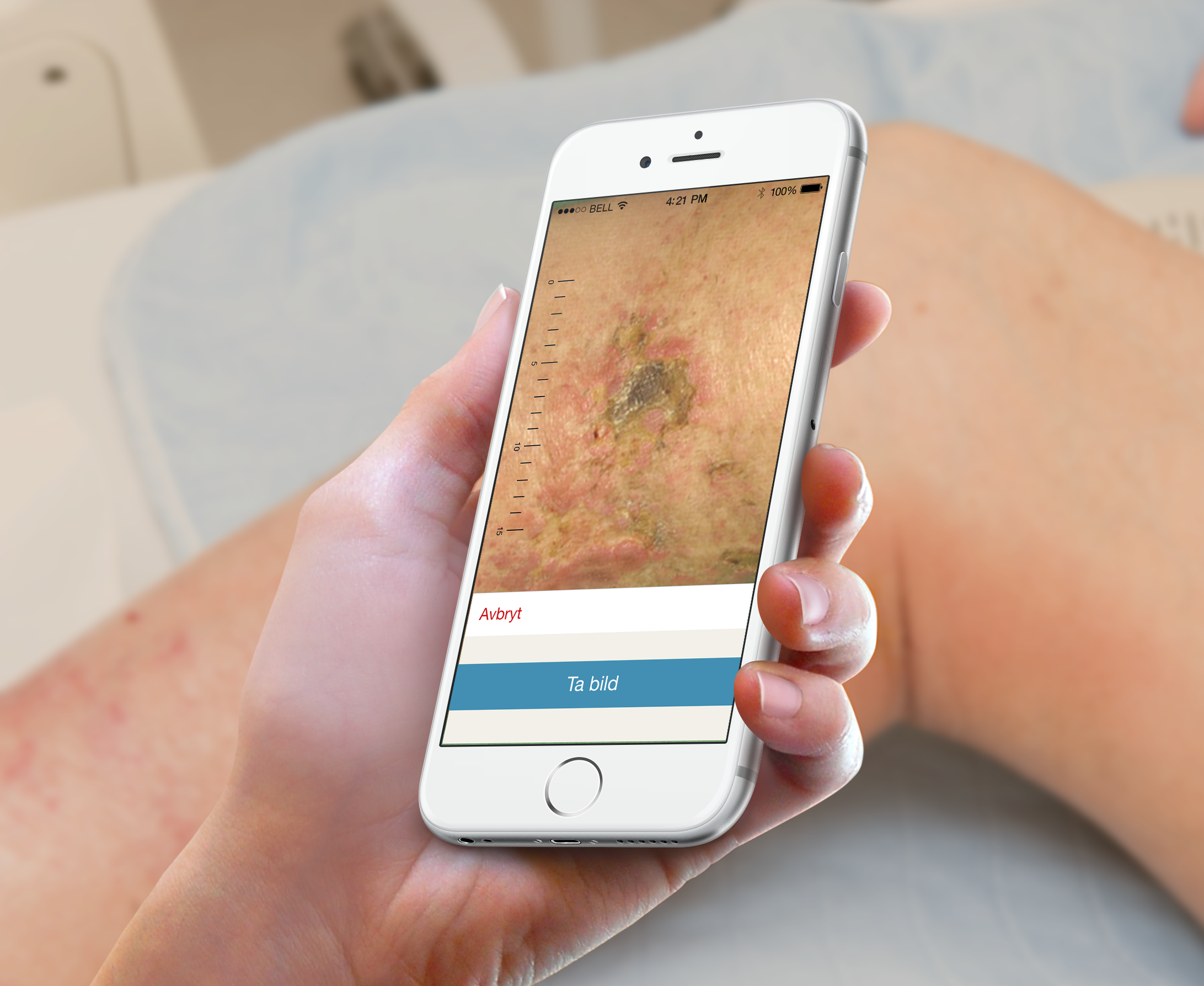

Supplement: Multimedia Appendix 1 [file humanfactors_v7i4e23188_app1.png]
